# Gender-specific differences in high-risk sexual behaviors among methamphetamine users in Myanmar-China border city, Muse, Myanmar: who is at risk?

**DOI:** 10.1186/s12889-018-5113-6

**Published:** 2018-02-01

**Authors:** Yu Mon Saw, Thu Nandar Saw, Nyein Chan, Su Myat Cho, Masamine Jimba

**Affiliations:** 1 Department of Healthcare Administration, Nagoya University Graduate School of Medicine, 65, Tsurumai-cho, Showa-ku, Nagoya, 466-8550 Japan; 2 Nagoya University Asian Satellite Campuses Institute, Nagoya, Japan; 3 Myanma Perfect Research, Yangon, Myanmar; 4 Department of Community and Global Health, Graduate School of Medicine, The University of Tokyo, Tokyo, Japan; 5 Department of Social Research, Defence Services Medical Research Centre, Tatkone Township, Nay Pyi Taw, Myanmar

**Keywords:** Sexual behavior, Gender, Methamphetamine, Drug users, Myanmar

## Abstract

**Background:**

Methamphetamine (MA) use is a significant public health concern due to its negative effects on health. However, to date, no epidemiological research has examined high-risk sexual behaviors (inconsistent condom use, having multiple sexual partners and having a history of sexually transmitted infections) among MA users. This topic is particularly important in Myanmar, which is recognized as one of the key MA production countries in the Southeast Asia region. Therefore, this study examined factors associated with high-risk sexual behaviors among MA users in Muse city, Myanmar.

**Methods:**

A community-based cross-sectional study was conducted from January to March 2013 in Muse city, Northern Shan State, Myanmar. In total, 1183 MA users (772 male; 411 female) were recruited using respondent-driven sampling and a computer assisted self-interviewing method. Generalized estimating equation models were used to examine factors associated with high-risk sexual behaviors.

**Results:**

A large proportion of MA users engaged in high-risk sexual behaviors (inconsistent condom use: males, 90.7%, females, 85.2%; multiple sexual partners: males, 94.2%, females, 47.2%; and history of STIs: males, 55.7%, females, 56.0%). Among males, being a multiple stimulants drug user (adjusted odds ratio [AOR] =1.77; 95% confidence interval [CI] =1.30–2.41) and being a client of sex workers (AOR = 1.41; 95% CI = 1.08–1.83) were risk factors for engaging in high-risk sexual behaviors. Among females, being a migrant worker (AOR = 2.70; 95% CI = 1.86–3.93) and being employed (AOR = 1.57; 95% CI = 1.13–2.18) were risk factors for engaging in high-risk sexual behaviors as well.

**Conclusions:**

High-risk sexual behaviors were particularly pronounced among both male and female MA users. MA prevention programs that reflect gender considerations should be developed to pay more attention to vulnerable populations such as migrants, clients of sex workers, and less educated female MA users.

## Background

Globally, methamphetamine (MA) use is a significant public health concern [1, 2]. Increased dependence on MA and its abuse are also associated with engagement in high-risk sexual behaviors [3–5]. This is because MA affects the central nervous system and can cause loss of inhibitory control over sexually compulsive behavior among its users. Thus, MA increases their engagement in risky sexual behaviors [3, 6]. For instance, compared to non-MA users, MA users visit sex workers more often, have more sexual partners, and use condoms less often [7, 8].

Moreover, MA use has been associated with the transmission of sexually transmitted infections (STIs) including human immunodeficiency virus (HIV), [9] and with unprotected anal sex among men who have sex with men (MSM). A number of possible causal mechanisms could underlie the positive correlation between MA use and high-risk sexual behaviors, assessed as engaging in unprotected sex, having multiple sex partners, and being diagnosed with an STI [8, 10]. First, some effects of MA may directly complement HIV risk behaviors. MA creates a sense of euphoria, pleasure, and sexual arousal while lowering inhibitions and clouding judgment. MA’s effects on judgment may lead to not using a condom even during high-risk sexual activity [11, 12]. Reflecting this, MA users participate in high-risk sexual behaviors more often when using MA. Second, other effects of MA may indirectly increase the likelihood of STI infection. While MA causes sexual arousal, it also impedes the person’s ability to achieve an erection [13, 14]. Thus, MSM who take MA may be more likely to engage in receptive anal sex [15, 16]. As such types of sexual intercourse are inherently more risky, MA users are more likely to be at risk of STI transmission [13]. Third, individuals who are addicted to MA, especially females, may resort to exchanging sex for money and/or drugs [17]. Finally, the positive correlation observed between MA use and high-risk sexual behaviors leading to STI transmission may be due to a higher preference for high-risk behaviors characteristic of those who use MA [18].

Changing risky sexual behaviors is an important strategy for preventing STI and HIV transmission among drug users [19]. Behaviors that can reduce the risk of STIs and HIV include regular testing and treatment for STIs, limiting the number of partners, and consistently using condoms for all sexual interactions; addressing all three prevention pillars is important for a comprehensive behavioral approach [20]. To this end, knowing which factors are associated with these behaviors among MA users is necessary to design effective prevention programs for this group. However, the studies investigating risky sexual behaviors among drug using populations focus on separate risk behavior measurements, such as only considering inconsistent condom use as the outcome variable. This kind of single risk behavior measurement may generate inaccurate or misleading results regarding sexual behavior-related risk among study participants. For example, individuals may increase their condom use while concurrently increasing their number of sexual episodes or partners [21]. Therefore, a comprehensive assessment is needed in order to identify the factors associated with overall sexual risk, for example by concurrently measuring inconsistent condom use, multiple sexual partners, and STI history concurrently [10].

To date, no epidemiological research has examined high-risk sexual behaviors (inconsistent condom use, having multiple sexual partners and having a history of STIs) among MA users in Myanmar. This topic is particularly important in Myanmar, which is recognized as one of the key MA production countries in the Southeast Asia region [18, 22]. The data regarding seizures of MA within Myanmar indicated national wide availability of MA. Myanmar youth start using MA in adolescence and the peak age of initiation of MA use is reached before 18 years. Many youths are also sexually active by this age. However, the Myanmar government cannot put much effort into controlling stimulant drug use because of the burden of opiate drug use that has long been a concern.

Regionally, Myanmar has the largest number of HIV-infected individuals among key populations [23] such as MSM, female sex workers (FSWs), people who inject drugs (PWID), and male STI patients [24]. These key populations also often use MA [8, 25–27]. HIV risk behaviors are known to be more pronounced among MA users [7]. However, the actual statistics on MA use and HIV prevalence among Myanmar’s MA users are not available. In view of the absence of information on this topic, the objective of the present study was to determine factors associated with high-risk sexual behavior among MA users in the Myanmar-China border city of Muse, Northern Shan State, Myanmar.

## Methods

A community-based cross-sectional study was conducted from January to March 2013 in Muse city, Northern Shan State, Myanmar. Being an MA user was defined as someone who had used MA at least three times in the 90 days prior to the interview. The study inclusion criteria were as follows: 1) self-reported MA users aged 18 to 35 years, 2) had used MA in the preceding three months, 3) had no withdrawal symptoms and were not under the influence of drugs at the time of interview, 4) had sex with any partner in the preceding six months, 5) able to read the Myanmar language, and 5) had provided informed consent for voluntary participation in the study.

A total of 1385 (782 males and 603 females) self-reported MA users were recruited by respondent-driven sampling (RDS) [28], using a computer assisted self-interviewing (CASI) method [29–31]. The CASI was conducted in private places and took 35–45 min to complete. MA users who had never had sexual intercourse or who did not answer at least one outcome measurement of sexual behaviors (11 males and 192 females) were excluded from the dataset. In total, 771 male and 441 female were included in the final data analysis.

### Respondent-driven sampling procedure

Participants were recruited using RDS method [28]. The sampling method is a variant of chain-referral sampling that utilizes an incentive for being interviewee and another incentive for recruiting their peers for interview. RDS is a widely used method in both developing and developed countries to recruit marginalized populations (also known as socially excluded populations) such as MSM, people who use drugs, and PWID [27, 32–37].

The RDS procedure was begun by selecting initial participants who were non-randomly selected members of the target population. A total of eight initial seeds (four males and four females) were interviewed and requested to recruit three other MA users to take part in this study. Each MA user was allowed to recruit up to three MA users to participate in the study within the two-week coupon expiration date. MA users received an initial incentive of 2000 Kyats (approximately $2.50) after completion of their own CASI session. They were eligible to receive the secondary incentive of a steel cup or lubricant gel that was equivalent to 900 Kyats if their referred MA user completed the CASI session. The other respondents were given the same opportunities as the first respondents for further recruitment and incentives. The details of the recruitment and interview procedures have been described elsewhere [33, 37].

### Measures

#### Dependent variables

To assess participants’ sexual risk behaviors, three HIV risk-related outcome variables for high-risk sexual behaviors were measured [19] using 24 items. In that questionnaires the participants’ sexual and condom use behaviors by partner types (regular, paid, exchanges, and casual), number of partners, and different sexual orientations (homosexual*,* heterosexual, or bisexual) were measured. For example, if one participant had more than two paid partners, the condom use behaviors of both paid partners were measured. Condom use was measured on a scale from 1 to 5 (always, almost, sometimes, never, and do not remember). The condom use variable by partner types were dichotomized into “consistent condom use: always” and “inconsistent condom use: almost, sometimes, never, and do not remember.” The most common STDs were measured using the locally used name in Myanmar such as chlamydia, gonorrhea, syphilis, HIB, genital warts, HIV, and other (with the request to specify). All three measures were asked with counter check (double filter) questions. Later, each measure was dichotomized and we recorded all the coding processes.

The high-risk sexual behaviors were defined as incorrect and/or inconsistent condom use across every incidence of sexual intercourse during the preceding six months, having had more than two sexual partners (i.e., multiple sex partners) during the preceding six months, and having ever been diagnosed with an STI (had past history of STIs and/or current infection) [10]. Such a composite variable of risky sexual behaviors is a more realistic method of examining risky sexual behavior than using a single risky behavior as an indicator. This is because participants might reduce their risk through one set of behaviors while increasing their risk in another set of behaviors, a phenomenon known as risk compensation. For example, participants might “compensate” for increasing their condom use by concomitantly increasing the frequency of intercourse or by increasing their number of sexual partners [21]. These variables were considered as high-risk sexual behaviors in this study under the following conditions: two direct measures (inconsistent condom use and multiple sexual partners in the preceding six months) and one indirect measure (history of STIs) [10]. The resulting variable was then dichotomized into “0” (no risk) and “1” (engaged in high-risk sexual behaviors) [10].

#### Independent variables

The assessed socio-demographic variables included current age, marital status, educational background, ethnic group, occupation, and current living status. Current age was used as a proxy for birth cohort (being born earlier or more recently) and divided into two groups (≤ 23 and > 23) based on the mean age of participants. Marital status was categorized as never married versus ever married. The participant’s educational background was created in terms of the formal education system of Myanmar: primary (1–5 years) and secondary (6–9 years) into secondary and below and high school (10–11 years), and university and higher (12 years of education or more) into High school or above. Ethnicity was grouped into the following categories: Shan, Kachin, Burma, or other (Kayar, Kayin, Chin, Mon, Rakkhin, multi-ethnicity, local residence of Indian and Chinese). Location was categorized as resident versus migrant, and employment status as unemployed versus employed.

MA use-related behaviors included age of MA initiation, ever-use of MA before and during sex, frequency of MA use, ever-use of more than two types of ATS, history of MA injection, and heroin used in the preceding six months were measured. The age of MA initiation was measured as continuous variable and later it was coded as a binary variable (< 17 versus ≤17), based on the average age of completion of high school at 17 years. Migrating to a new environment may play an important role in initiation and continuation of illegal substance and risky sexual behavior. Therefore, a variable was created to define residency status of the participants and categorized as permanent resident versus migrant. Additionally, a binary variable was created to define whether the respondents had ever been imprisoned, and a continuous measure was used to define age at sexual initiation. Sexual orientation and a visit to sex workers in the preceding six months were considered only for male MA users because of the low response of female users.

### Statistical analysis

The data were analyzed using Statistical Package for the Social Sciences (SPSS) version 18 (SPSS Inc., Chicago, IL, USA). Descriptive analyses were conducted to estimate the frequencies of the indicators of high-risk sexual behaviors and the frequencies of the other covariates, and to determine whether they differed between the male and female MA users. In all analyses, the level of significance was set at *p* <  0.05.

The three multiple logistic regression models were performed by gender to analyze three indicators of high-risk sexual behavior [inconsistent condom use, multiple sexual partners in the preceding six months and had a history of STIs (past and/or current)]. After that, the three indicators of high-risk sexual behavior were categorized into a single dichotomized variable to take into account the repeated inferential statistical tests for the three indicators of risky sexual behaviors.

Furthermore, the three indicators of high-risk sexual behavior assessed in this study tend to be correlated for any given individual [10]. The generalized estimating equation (GEE) model approach was therefore applied to accommodate these interrelationships. The GEE logistic regression was used to detect differences in the profiles of high-risk sexual behaviors across the male and female groups as well as the groups defined via other covariates. This methodology is similar to that used to examine multiple indicators of high-risk sexual behavior [10] and drug dependence symptoms [38]. This study used unweighted data although the personal network size of study participant was measured and the RDS sampling method was used. The covariates were tested for multicollinearity before being simultaneously fitted in the model.

## Results

### Descriptive statistics

The total of number of participants was 1183, with 772 male and 411 female participants. A statistically significant difference was observed between males and females regarding their age (*p* <  0.001; Table 1), with 35.2% of males and 32.8% of females being aged between 21 and 24 years (Table 1). With regard to education, 39.4% of males and 47.7% of females had at least a secondary level of education (*p* <  0.001), and 24.2% of males and 38.4% of females were unemployed (*p* <  0.001). Males were more likely than females to be single (71.2% versus 67.4%). Regarding ethnicity, females were significantly more likely than males to belong to the Shan (36.3% versus 27.5%) or Burmese (33.8% versus 28.1%) ethnic groups. In addition, a significantly higher proportion of both males (56.9%) and females (69.8%) had migrated from another part of Myanmar (*p* <  0.001) as opposed to being native to the region.

**Table 1:** Socio-demographic characteristics of participants (*N* = 1183)

Characteristics	Total (N = 1183)		Male (n = 772)		Female (n = 411)		*P*-value
	N	%	n	%	n	%	
Age							0.004
≤ 20	288	24.3	163	21.1	125	30.4	
21–24	407	34.4	272	35.2	135	32.8	
25–28	361	30.5	249	32.3	112	27.3	
≤ 29	127	10.7	88	11.4	39	9.5	
Marital status							0.170
Never married	827	69.9	550	71.2	277	67.4	
Ever married	356	30.1	222	28.8	134	32.6	
Education							< 0.001
Primary school	117	9.9	54	7.0	63	15.3	
Secondary school	500	42.3	304	39.4	196	47.7	
High school	274	23.2	208	26.9	66	16.1	
University	292	24.7	206	26.7	86	20.9	
Ethnicity							< 0.001
Shan	361	30.5	212	27.5	149	36.3	
Kachin	229	19.4	157	20.3	72	17.5	
Burma	356	30.1	217	28.1	139	33.8	
Others	237	20.0	186	24.1	51	12.4	
Employment status							< 0.001
Unemployed	345	29.2	187	24.2	158	38.4	
Employed	838	70.8	585	75.8	253	61.6	
Living status							< 0.001
Resident	457	38.6	333	43.1	124	30.2	
Migrant	726	61.4	439	56.9	287	69.8	

### Prevalence of risky sexual behaviors

The findings revealed that a large proportion of MA users engaged in high-risk sexual behaviors (inconsistent condom use: males, 90.7%, females, 85.2%; multiple sexual partners: males, 94.2%, females, 47.2%; and history of STIs: males, 55.7%, females, 56.0%).

### Multivariate analysis

The data revealed that employed males (adjusted odds ratio [AOR] =1.42; 95% confidence interval [CI] =1.08–1.87), and male MA users who used MA before and during sex (AOR = 1.67; 95% CI = 1.23–2.28), were more likely to use condoms inconsistently, to have had multiple sexual partners in the preceding six months and to have an STI history (for all three high-risk sexual behaviors; Table 2). Compared to the male participants who had not visited sex workers in the preceding six months, those who visited sex workers in the preceding six months were more likely to engage in high-risk sexual behaviors (AOR = 1.41, 95% CI = 1.08–1.83). In addition, male MA users who used more than two types of ATS (AOR = 1.77; 95% CI = 1.30–2.41) were more likely to be involved in high-risk sexual behavior.

**Table 2:** Generalized estimating equations for high-risk sexual behaviors^a^ of methamphetamine users by gender

	Male (n = 771)^b^				Female (n = 411)^c^			
	n	%	AOR	95% CI	n	%	AOR	95% CI
Age (current age at survey)								
≤ 23	435	56.4			260	63.3		
> 23	336	43.6	1.28	0.96–1.70	151	36.7	1.15	0.79–1.68
Marital status								
Never married	549	71.2			277	67.4		
Ever married	222	28.8	1.26	0.95–1.67	134	32.6	1.10	0.78–1.54
Education								
Secondary school and below	357	46.3			259	63.0		
High school or above	414	53.7	0.98	0.77–1.24	152	37.0	0.42	0.31–0.56
Ethnicity								
Shan	212	27.5			149	36.3		
Kachin	158	20.5	1.17	0.85–1.60	72	17.5	0.72	0.43–1.19
Burma	215	27.9	1.07	0.78–1.49	139	33.8	1.24	0.75–2.05
Others	186	24.1	0.91	0.65–1.29	51	12.4	1.48	0.95–2.31
Employment status								
Unemployed	188	24.4			158	38.4		
Employed	583	75.6	1.42	1.08–1.87	253	61.6	1.57	1.13–2.18
Living status								
Resident	336	43.6			124	30.2		
Migrant	435	56.4	1.13	0.89–1.45	287	69.8	2.70	1.86–3.93
Age of sexual initiation								
> 17	226	29.3			221	53.8		
≤ 17	545	70.7	0.95	0.74–1.21	190	46.2	1.02	0.77–1.34
Sexual orientation								
Hetero-sexual	327	42.4						
Bi/homo-sexual	444	57.6	1.15	0.89–1.48	S/S			
Had visited sex worker within six months								
No	220	28.5						
Yes	551	71.5	1.41	1.08–1.83	S/S			
Age of methamphetamine initiation								
> 17	208	27.0			177	43.1		
≤ 17	563	73.0	0.85	0.65–1.12	234	56.9	0.95	0.73–1.24
Had used methamphetamine before and/or during sex								
No	96	12.5			130	31.6		
Yes	675	87.5	1.67	1.23–2.28	281	68.4	3.39	2.51–4.56
Frequency of methamphetamine use^d^								
> 3 times a month	160	20.8			98	23.9		
Once a week	223	28.9	1.31	0.95–1.81	101	24.6	2.06	1.41–3.02
> 4 times a week	193	25.0	1.23	0.91–1.67	83	20.2	1.37	0.95–1.99
≤ 4 times a week	195	25.3	1.29	0.95–1.77	128	31.3	2.44	1.66–3.60
Type of amphetamine*-*type stimulant used within three months								
Only methamphetamine	591	12.5			409	99.5		
Multiple stimulants	180	87.5	1.77	1.30–2.41	2	0.5		
History of injection use of methamphetamine								
No	647	83.9			345	83.9		
Yes	124	16.1	1.12	0.76–1.64	66	16.1	1.40	0.90–2.20
Had used heroin in the preceding six months								
No	638	82.7			295	71.8		
Yes	133	17.3	1.01	0.70–1.45	116	28.2	1.10	0.78–1.55
Ever been to prison								
No	741	96.1			397	96.6		
Yes	30	3.9	0.93	0.52–1.69	14	3.4	0.54	0.27–1.07

Note. *AOR* Adjusted odd ratio, *CI* Confidence interval, *S/S* Small sample size. ^d^One female participant did not respond to this question

^a^High-risk sexual behaviors measured were incorrect and/or inconsistent condom use across every incidence of sexual intercourse in the preceding six months, having had more than two partners (had multiple sex partners) within the preceding six months, and having ever been diagnosed with an STI (past and/or current)

^b^Adjusted for age, marital status, education, ethnicity, employment status, living status, age of sexual initiation, sexual orientation, had visited sex worker within six months, age of methamphetamine initiation, had used methamphetamine before and during sex, frequency of methamphetamine use, had used more than two types of ATS, history of injection use of methamphetamine, had used heroin in the preceding six months, and ever been to prison

^c^Adjusted for age, marital status, education, ethnicity, employment status, living status, age of sexual initiation, age of methamphetamine initiation, had used methamphetamine before and during sex, frequency of methamphetamine use, history of injection use of methamphetamine, had used heroin in the preceding six months, and ever been to prison

Meanwhile, female participants who had high school or higher level of education (AOR = 0.42, 95% CI = 0.31–0.56) were less likely to engage in high-risk sexual behaviors compared to those with secondary or below level of education. The employed female participants were also more likely to engage in high-risk sexual behaviors compared to their counterparts who were not employed (AOR = 1.57; 95% CI = 1.13–2.18). Moreover, participants who migrated from other parts of Myanmar (AOR = 2.70; 95% CI = 1.86–3.39) were more likely to engage in high-risk sexual behaviors compared to non-migrant female participants. Furthermore, those female participants who had used MA before and during sex (AOR = 3.39, 95% CI = 2.51–4.56) and who used MA once a week (AOR = 2.06, 95% CI = 1.41–3.02) or more than four times a week (AOR = 2.44, 95% CI = 1.66–3.60) were more likely to engage in high-risk sexual behaviors.

## Discussion

This study revealed the alarming extent to which MA users are engaged in high-risk sexual behaviors in the Myanmar-Chinese border city of Muse. Further, both males and females who used MA before and during sex and who were employed at the time of the survey were more likely to use condoms inconsistently, to have had multiple sexual partners within the preceding six months, and to have a history of STIs. Among male MA users, those who used more than two types of ATS and those who had visited a sex worker in the preceding six months were more likely to be involved in high-risk sexual behaviors for all three high-risk sexual behaviors. Among female MA users, those who had an education level of high school or above were less likely than women with a secondary or below level of education to use condoms inconsistently, to have had multiple sexual partners within the preceding six months, and to have an STI history. Female MA users who were migrants, who used MA at least four times per week, and who used MA once a week were more likely to be involved in high-risk sexual behaviors.

Among both male and female participants, using MA before and during sexual intercourse was associated with a greater likelihood of engagement in high-risk sexual behaviors. This is understandable, as MA use causes loss of inhibitory control leading to sexually compulsive behaviors, and can cloud judgment [3, 6]. Therefore, its use before or during sexual intercourse may lead to sexual intercourse without condom use and other risky sexual behaviors [3, 6, 39–41]. Previous studies have similarly identified MA use before and during sexual activities as one a significant risk factor for decreasing the use of condoms during vaginal and anal intercourse among PWID, MSM, and FSWs [7, 42]. In this study, MA use before and during sexual activities was associated with inconsistent condom use, having multiple sexual partners within the preceding six months, and having a history of STIs. Thus, MA use before or during intercourse may further escalate the STI and HIV epidemic.

In this study, female MA users who used MA more than four times a week and those who used MA once a week were more likely to be involved in high-risk sexual behavior. MA is an extremely addictive drug and long-term use can result in sexual health problems such as STIs and HIV [22, 42, 43]. This kind of heavy and regular MA use can lead to failure to control high-risk sexual behaviors such as unprotected sexual intercourse. It also prolongs sexual activities, thus increasing the risk of HIV and STI transmission [44, 45]. This may be due to the fact that MA use increases sexual desire, arousal, and pleasure [39–41, 44]. These findings are also in line with previous observations, which have shown that heavier or greater frequency of drug use has a multitude of biological and cognitive effects that may increase sexual risk-taking behaviors [46]. Furthermore, a review study found that drug users who engage in more high-risk sexual behaviors tend to be heavier drug users than those who engage in fewer high-risk sexual behaviors [47].

Beyond factors related to MA use, being employed was positively associated with high-risk sexual behaviors among both male and female MA users. One possible explanation for this finding is that individuals who have jobs might have more opportunities to engage in dyadic relations through their professional and personal lives [48, 49]. Similar results were reported from Thailand [50], where it was shown that paid workers were more likely to use addictive substances and to be engaged in sexual risk behaviors. A study in Bangladesh also found that paid female jobholders had a greater tendency to engage in sexual risk behaviors [51].

Male participants who had visited sex worker in the preceding six months were also more likely to engage in high-risk behaviors. This may be because male participants who had visited sex workers may use MA to enhance their sexual activities [46, 47, 52]. In the United States, MA users have been shown to visit sex workers more often than non-MA users, and to engage in unprotected sex more frequently [7]. The association between drug use and high-risk sexual behavior is also pronounced among male MA users [46, 47, 52].

Furthermore, they are more likely to contract HIV and other STIs [53, 54]. The use of more than two types of ATS was positively associated with high-risk behaviors among male MA users [55]. The association between the use of multiple stimulants and high-risk sexual behaviors is particularly pronounced among youth and adult populations and especially among MSM [56–58]. Distinct from other illicit drugs, stimulant drugs encourage its users to engage in prolonged sexual activities which may lead to higher rates of unprotected anal intercourse [7, 59]. Ultimately, it may also lead to greater risk for contracting HIV and other STIs [53, 54].

A high education level among female MA users was negatively associated with high-risk sexual behaviors. The probability of reporting high-risk sexual behaviors decreased with increasing educational level in GEE analyses. Similar findings were reported for Kenyan females, with each increase in education level associated with an increased report of condom use with last partner [60]. However, past STI diagnosis was negatively associated with junior high school education compared to a primary or lower level education among Chinese FSWs [61]. Nevertheless, the findings of the current study are supported by research performed elsewhere, [62] and suggest that prevention programs should pay greater attention to less educated female MA users in order to reduce high-risk sexual behavior.

Notably, migrant females were more likely to engage in high-risk sexual behaviors compared to non-migrant users in the present study. This association did not reach a statistically significant level among male migrant MA users. Several reasons may account for these differences in association. First, in this study, one of the risk behavior measurements was having had a history of STIs, and females have a higher biological susceptibility than males to STI and HIV infection [63]. For example, females have been estimated to have two to four times higher chance of being infected with HIV through unprotected vaginal sex with an HIV-infected partner than males [63]. Second, sociological tendencies toward an imbalance of power in relationships and socioeconomic dependency on males may impair females’ decision-making about condom use [64, 65]. These factors may further increase the risk of contracting an STI among migrant drug users. Third, the working environment may render female migrants more susceptible to increased MA use at the workplace. Notably, the study area of Muse is located near the Chinese border, and female migrants often work at the nightclubs and bars in this location. This kind of work requires them to stay awake for the whole night and therefore might increase their vulnerability to sexual violence, risky sexual behaviors, and subsequent STI transmission [66, 67].

Several methodological limitations should be acknowledged in interpreting the results of this study. First, the study was retrospective in nature and therefore some information may have been forgotten or lost by the respondents. Second, a social desirability bias could also affect responses about sexual behavior. However, to minimize this problem, several strategies were undertaken. For example CASI was used to administer the survey. Third, the dataset was not weighted, although RDS sampling method was applied. Finally, this study is cross-sectional in design and therefore does not allow us to establish a causal relationship between MA use and risky sexual behaviors. Additionally, consumption of MA might be associated with other characteristics of the participants, such as antisocial personality traits, sensation-seeking behavior, or mental disorders, and accounting for these potential covariates might better explain the observed association [68, 69]. Despite such limitations, our findings have important implications for better understanding the risk factors for high-risk sexual behavior among both male and female MA users and thereby can help toward in the development of new STI and HIV prevention strategies.

## Conclusion

The current study contributes valuable information about high-risk sexual behaviors among MA users in Muse, Myanmar. A large proportion of both male and female MA users engaged in inconsistent condom use in the preceding six months, had multiple sexual partners within the preceding six months, and had a history of STIs. Both male and female MA users before and during sex were more likely to engage in high-risk sexual behaviors, which may in turn increase the possibility of spreading STIs, including HIV within this population and to their partners. Comprehensive and targeted MA prevention strategies and programs should give greater attention to employed MA users and vulnerable populations such as migrants, clients of sex workers, and less educated female MA users.

## Abbreviations

AOR: Adjusted odds ratio
ATS: Amphetamine-type stimulants
CASI: Computer assisted self-interviewing
CI: Confidence interval
FSWs: Female sex workers
GEE: Generalized estimating eq.
HIV: Human immunodeficiency virus
MA: Methamphetamine
MSM: Men who have sex with men
PWID: People who inject drugs
RDS: Respondent-driven sampling
SPSS: Statistical Package for the Social Science
STIs: Sexually transmitted infections
